# Maternal mortality and its relationship to Emergency Obstetric Care (EmOC) in a tertiary care hospital in South India

**DOI:** 10.1186/1865-1380-7-S1-O7

**Published:** 2014-07-25

**Authors:** Papa Dasari

**Affiliations:** 1 Department of Obstetrics & Gynecology, Jawaharlal Institute of Postgraduate Medical Education and Research, Puducherry, India

## Objective

To determine the trends in maternal mortality ratio for the past 5 years at JIPMER Hospital and to find out the proportion of maternal deaths in relation to emergency admissions.

## Methods

Setting: tertiary care teaching hospital.

Study design: retrospective analysis.

## Results

There were 106 maternal deaths among 73,935 live births; one death was misclassified as maternal death and record of one maternal death was unavailable. The overall MMR was 142 per 100000 live births. The MMR for 2008, 2009, 2010, 2011 and 2012 was 104, 180, 187, 115 and 157 respectively. The trend is increasing proportion of indirect causes from 2008 to 2012. Of the direct causes, haemorrhage was the leading cause followed by hypertensive disorders and sepsis. Among the indirect causes heart disease was the leading cause followed by severe anaemia and jaundice complicating pregnancy. Of the 104 maternal deaths 95 (90%) were emergency admissions and 56 (59%) of them were referred. Thirty two percent of them died within 24 hours of admission. Thirty six percent of maternal deaths were associated with still births especially macerated intrauterine deaths (26%) and 8.7% died undelivered.

## Conclusion

There is an increasing trend of indirect causes of maternal death from 2008 to 2012. Overall, 40% of maternal deaths were due to indirect causes as against the National average of 20%. Ninety percent of maternal deaths were emergency admissions coming with complications. Though the MMR is less than the National/ Global average, efforts are required to achieve the MDG goal of 100 by 2015.

